# Nitrogen dynamics in Turbic Cryosols from Siberia and Greenland^☆^

**DOI:** 10.1016/j.soilbio.2013.08.004

**Published:** 2013-12

**Authors:** Birgit Wild, Jörg Schnecker, Jiří Bárta, Petr Čapek, Georg Guggenberger, Florian Hofhansl, Christina Kaiser, Nikolaj Lashchinsky, Robert Mikutta, Maria Mooshammer, Hana Šantrůčková, Olga Shibistova, Tim Urich, Sergey A. Zimov, Andreas Richter

**Affiliations:** a University of Vienna, Department of Microbiology and Ecosystem Science, Division of Terrestrial Ecosystem Research, Althanstrasse 14, 1090 Vienna, Austria; b Austrian Polar Research Institute, 1090 Vienna, Austria; c University of South Bohemia, Department of Ecosystems Biology, Branišovská 31, 37005 České Budějovice, Czech Republic; d Leibniz Universität Hannover, Institut für Bodenkunde, Herrenhäuser Strasse 2, 30419 Hannover, Germany; e International Institute for Applied Systems Analysis (IIASA), Schlossplatz 1, 2361 Laxenburg, Austria; f Central Siberian Botanical Garden, Siberian Branch of Russian Academy of Sciences, St. Zolotodolinskaya 101, 630090 Novosibirsk, Russia; g VN Sukachev Institute of Forest, Siberian Branch of Russian Academy of Sciences, Akademgorodok, 660036 Krasnoyarsk, Russia; h University of Vienna, Department of Ecogenomics and Systems Biology, Althanstrasse 14, 1090 Vienna, Austria; i University of Bergen, Department of Biology/Centre for Geobiology, Allégaten 41, 5007 Bergen, Norway; j Northeast Scientific Station, Pacific Institute for Geography, Far-East Branch of Russian Academy of Sciences, 678830 Chersky, Republic of Sakha, Russia

**Keywords:** Arctic, Tundra, Cryoturbation, Soil organic matter, Ecological stoichiometry, Nitrogen transformation, Protein depolymerization, Nitrogen mineralization, Nitrification, Nitrogen availability

## Abstract

Turbic Cryosols (permafrost soils characterized by cryoturbation, i.e., by mixing of soil layers due to freezing and thawing) are widespread across the Arctic, and contain large amounts of poorly decomposed organic material buried in the subsoil. This cryoturbated organic matter exhibits retarded decomposition compared to organic material in the topsoil. Since soil organic matter (SOM) decomposition is known to be tightly linked to N availability, we investigated N transformation rates in different soil horizons of three tundra sites in north-eastern Siberia and Greenland. We measured gross rates of protein depolymerization, N mineralization (ammonification) and nitrification, as well as microbial uptake of amino acids and NH_4_^+^ using an array of ^15^N pool dilution approaches. We found that all sites and horizons were characterized by low N availability, as indicated by low N mineralization compared to protein depolymerization rates (with gross N mineralization accounting on average for 14% of gross protein depolymerization). The proportion of organic N mineralized was significantly higher at the Greenland than at the Siberian sites, suggesting differences in N limitation. The proportion of organic N mineralized, however, did not differ significantly between soil horizons, pointing to a similar N demand of the microbial community of each horizon. In contrast, absolute N transformation rates were significantly lower in cryoturbated than in organic horizons, with cryoturbated horizons reaching not more than 32% of the transformation rates in organic horizons. Our results thus indicate a deceleration of the entire N cycle in cryoturbated soil horizons, especially strongly reduced rates of protein depolymerization (16% of organic horizons) which is considered the rate-limiting step in soil N cycling.

## Highlights

• N turnover in cryoturbated Arctic soils measured by isotope pool dilution assays.
• Protein depolymerization strongly exceeds NH_4_^+^ and NO_3_^−^ production rates.
• Microbial N use efficiency indicates N limitation in Siberian sampling sites.
• Deceleration of microbial N transformation rates by cryoturbation.

## 1 Introduction

Arctic soils are commonly affected by cryoturbation, i.e., by a mixing of soil layers due to freeze–thaw processes, and are thus often characterized by horizons of poorly decomposed material subducted into and surrounded by mineral subsoil (Bockheim, 2007; Tarnocai et al., 2009). Cryoturbated soils are estimated to store 581 Gt of organic carbon that is currently protected from fast decomposition (Tarnocai et al., 2009). Although cryoturbated horizons (Ojj or Ajj) are chemically similar to organic (O) or mineral topsoil (A) horizons, they are usually several hundred to thousand years older (Xu et al., 2009; Hugelius et al., 2010), suggesting that C mineralization is slowed down in cryoturbated horizons. Likewise, N mineralization has been found to be lower in cryoturbated than in all other horizons, i.e., in organic (O), mineral topsoil (A), and mineral subsoil (B) horizons (Kaiser et al., 2007). Cryoturbation thus lead to retardation of soil organic matter (SOM) decomposition in general, and of N mineralization in particular, and this retardation cannot be explained by lower subsoil temperatures alone (Kaiser et al., 2007).

The rate limiting step for soil N cycling is the breakdown of N-rich, high molecular weight organic compounds by extracellular enzymes, especially the depolymerization of proteins (Schimel and Bennett, 2004; Geisseler et al., 2010; Jones and Kielland, 2012). The resulting oligopeptides and amino acids are taken up by microorganisms and are further mineralized to NH_4_^+^ which, in turn, is the substrate for nitrification (Jones and Kielland, 2012). Nitrogen mineralization is considered an overflow mechanism, i.e., only an excess of N that cannot be used to build up biomass (because another element is limiting) will be mineralized (Schimel and Bennett, 2004). Thus, gross N mineralization rates are expected to increase with increasing N availability, and, consequently, microbial N use efficiency (NUE; the proportion of N taken up that is incorporated into microbial biomass) will decrease.

Nitrogen availability in high latitude ecosystems is generally low (Schimel and Bennett, 2004), but is predicted to increase with global warming. Higher soil temperatures were shown to increase soil microbial activity, resulting in increased net N mineralization, and thus N availability (Nadelhoffer et al., 1991; Hobbie, 1996; Rustad et al., 2001). Additionally, the functional composition of tundra vegetation is expected to shift (Elmendorf et al., 2012), which is likely to alter the availability of N for soil microorganisms by changing patterns in plant N uptake, litter decomposability and competition for N in the rhizosphere (Wookey et al., 2009). An increase in N availability can stimulate plant and microbial growth (e.g., Hobbie et al., 2002; Sistla et al., 2012), and also SOM decomposition. In a long-term fertilization study in Alaskan tundra, Mack et al. (2004) observed that fertilization not only increased plant production, but also SOM decomposition, overall resulting in a net loss of C from the system. This suggests that the emission of CO_2_ from Arctic soils could be amplified by increased N availability. The effect of N additions on SOM decomposition is, however, highly variable. Across sites, and across soil horizons, both stimulation and inhibition of SOM decomposition by N addition have been observed (Lavoie et al., 2011). To predict the overall effects of N availability on SOM decomposition in Arctic soils in a future climate, an in-depth understanding of all steps of N cycling in the soil profile is necessary. So far most studies have exclusively focused on N mineralization and nitrification, but the depolymerization step of highmolecular weight organic N, which has been suggested to be rate-limiting, has not received much attention.

While many studies have investigated N dynamics in organic and mineral topsoil horizons, not much is known about mineral and cryoturbated horizons in the subsoil. In spite of the large amounts of poorly decomposed SOM in cryoturbated horizons, and in spite of the importance of N for SOM decomposition, we are only aware of one study investigating N dynamics in cryoturbated horizons. This study showed a reduction in N mineralization rates in cryoturbated compared to organic horizons (Kaiser et al., 2007). These low N mineralization rates could either have been the consequence of reduced protein depolymerization rates, or of a higher N demand of the microbial community. In the latter case, protein depolymerization would be similar in all horizons, but N mineralization would be lower in cryoturbated horizons, indicating that the microbial community needed a higher proportion of the available N for growth, and thus had a higher NUE.

We here report on microbial N transformations and NUE across different horizons in the active layer of tundra soil. We hypothesized that the observed reduction of gross N mineralization in cryoturbated horizons compared to organic horizons was due to a reduction in protein depolymerization, not due to a higher NUE of the microbial community. Since the capacity to depolymerize proteins constitutes a property of the microbial community, we further investigated the microbial community structure in different soil horizons, with the goal to identify groups that may be responsible for individual N transformations.

To achieve these goals, we sampled Turbic Cryosols (Turbels) at three sites in Siberia and Greenland and measured gross rates of protein depolymerization, microbial amino acid uptake, N mineralization, NH_4_^+^ uptake and nitrification using an array of ^15^N pool dilution approaches. Additionally, we estimated the microbial community composition using phospholipid fatty acids (PLFAs) as biomarkers.

## 2 Material & methods

### 2.1 Sampling sites

We compared soils from three different sites: (1) The heath tundra site was located in eastern Greenland close to the Zackenberg Research Station (typical tundra subzone; 74°29′ N, 20°32′ W) on sedimentary bedrock (sandstone). It was dominated by *Cassiope tetragona*, *Vaccinium uliginosum*, *Dryas octopetala*, *Salix arctica*, and *Carex* sp., with lichens between the dwarf shrubs. (2) The shrub tundra site was in north-eastern Siberia close to the town of Chersky (southern tundra subzone; 68°45′ N, 161°36′ E) on aeolian late Pleistocene sediment. It was located in a shrubby moss lichen tundra dominated by *Betula exilis*, *Vaccinium uliginosum*, *Flavocetraria nivalis*, *Flavocetraria cucullata* and *Aulacomnium turgidum*, with sparse *Larix gmelinii* trees. (3) The tussock tundra site was approximately 80 km north of Chersky (southern tundra subzone; 69°26′ N, 161°44′ E) on aeolian late Pleistocene sediment. It was dominated by *Eriophorum vaginatum*, *Carex lugens, B. exilis*, *Vaccinium vitis-idaea*, *Aulacomnium turgidum*, and *Dicranum* sp.

All soils were classified as Turbic Cryosols according to the World Reference Base for Soil Resources (IUSS Working Group WRB, 2007) or Turbels according to the US Soil Taxonomy (Soil Survey Staff, 1999). The active layer at the time of sampling was 47 cm (heath tundra, Greenland), 73 cm (shrub tundra, Siberia; under frost boils) and 72 cm (tussock tundra, Siberia; under frost boils).

At each site, we took samples from the active layer of three replicate soil pits. We sampled organic layers (O, including OA horizons) from the soil surface (further termed “organic horizon”). We then took samples from pockets of organic material (Ojj) or mineral topsoil (Ajj) that was buried within the subsoil. Such buried horizons, caused by cryoturbations, i.e., by subduction of organic material or mineral topsoil into the subsoil due to freeze–thaw processes (Bockheim, 2007), are common in permafrost soils. Sampled pockets of cryoturbated material were between 20 and 50 cm from the soil surface (further termed “cryoturbated horizon”). We further sampled the mineral soil surrounding the cryoturbated pockets (A, AB, B or Cg horizons, at depths from 10 to 60 cm from the surface). These horizons are collectively termed “mineral horizon”. Living roots were carefully removed and samples were kept cool until analysis.

All samplings took place in August 2010. We are aware of the fact that N cycling and N availability show seasonal variation in Arctic soils (e.g., Weintraub and Schimel, 2005a,b; Edwards et al., 2006). Data presented here do not reflect these variations, but represent N cycling in the late growing season.

### 2.2 Basic characterization and nutrient concentrations

Concentrations of ammonium and nitrate were determined photometrically in 1 M KCl extracts following Kandeler and Gerber (1988) and Miranda et al. (2001), respectively. Total dissolved nitrogen (TDN) was measured with a DOC/TN analyzer (Elementar LiquiToc II or Shimadzu TOC-V_CPH/CPN_/TNM-1) in 0.5 M K_2_SO_4_ or 1 M KCl extracts (samples from Siberia and Greenland, respectively), and dissolved organic N (DON) was calculated by subtracting NH_4_^+^ and NO_3_^−^ from TDN. Extraction with K_2_SO_4_ or KCl was previously shown to yield similar DON recovery (Jones and Willett, 2006). For determination of inorganic phosphate, soil samples were extracted with 0.5 M NaHCO_3_ (Olsen et al., 1954) and measured photometrically with the molybdate-ascorbic acid method (Murphy and Riley, 1962). Contents of organic C and total N were determined in dried and ground samples using elemental analysis-isotope ratio mass spectrometry (EA-IRMS), either with a CE Instrument EA 1110 elemental analyzer coupled to a Finnigan MAT DeltaPlus IRMS with a Finnigan MAT ConFlo II Interface, or with an Isoprime EA-IRMS system. Samples from Siberia contained traces of carbonate and were acidified in HCl atmosphere and neutralized over NaOH before EA-IRMS analysis. For determination of total P content, samples were amended with a mixture of concentrated HClO_4_ and HNO_3_ (1:4), heated stepwise to 160 °C and 220 °C for digestion, cooled to room temperature, filtered (Whatman 40 ashfree cellulose filter) and measured with inductively coupled plasma-optical emission spectrometry (ICP-OES, Perkin Elmer Optima 3000 XL) against external standards. Ratios of C, N and P were calculated as mass ratios. pH values were determined in suspensions of dried soil in de-ionized water (1:2.5; weight:volume).

### 2.3 Gross rates of N transformations

Gross rates of protein depolymerization and amino acid uptake were determined using a ^15^N pool dilution assay as described by Wanek et al. (2010), with slight modifications to account for the low amino acid concentrations in soils. Briefly, a mixture of 20 ^15^N labeled amino acids (>98 at%, Spectra and Cambridge Isotope Laboratories) was dissolved in 10 mM CaSO_4_ and added to 2 g of field-moist soil in duplicates. Per sample, 500 μl solution containing 2.5 μg total amino acids were applied. After incubation for 10 or 30 min at 7 °C, activities were stopped with 19.5 ml of 10 mM CaSO_4_ containing 3.7% of formaldehyde. Samples were extracted for 5 min and either allowed to settle for 10 min (both Siberian sites) or centrifuged for 5 min at 10 845 g (Greenland heath tundra site). Samples were filtered through synthetic wool and GF/C filters (Whatman), and loaded on cation exchange cartridges (OnGuard II H 1cc cartridges, Dionex, cleaned with 3 M ammonia and re-generated with 1 M HCl before use). After application of the samples, cartridges were washed with 10 ml distilled water, stabilized with 5 ml 5% methanol and stored cool until elution. With each batch of samples, blanks and amino acid standards were processed to correct for losses due to ion exchange. After elution of the amino acids from the cartridges with 30 ml 3 M ammonia, an internal standard was added to the samples (1 μg of nor-valine, nor-leucine and para-chloro-phenylalanine each, Sigma–Aldrich). Samples were dried with rotary evaporation, re-dissolved in 1.5 ml 20% ethanol and dried in a SpeedVac system. Finally, samples were derivatized with chloroformate before analysis with a gas chromatography–mass spectrometry system (GC–MS), consisting of a CTC autosampler (CTC Analytics) and a Trace GC Ultra coupled to a quadrupole mass spectrometer (DSQ II; Thermo Scientific). Two μl of sample were injected in splitless mode (injector temperature 270 °C), separated on an Equity-1701 column (30 m × 0.25 mm × 1 μm; Sigma–Aldrich) with 1 ml min^−1^ He as carrier gas (GC method: 105 °C for 1 min, 6 °C min^−1^ to 135 °C, 3 °C min^−1^ to 180 °C, 20 °C min^−1^ to 260 °C, 260 °C for 35 min) and detected in Selected Ion Monitoring mode. Concentrations of alanine, valine, leucine, isoleucine, proline, tryptophane, phenylalanine and tyrosine were calculated using calibrations against external standards, and the abundance of ^15^N in each of these amino acids was calculated based on peak areas of fragments characteristic for ^14^N and ^15^N (for fragments see Wanek et al., 2010), using a calibration against standards of different ^15^N abundance.

Gross rates of N mineralization (ammonification), NH_4_^+^ uptake and nitrification were determined as described by Kaiser et al. (2011) by adding 500 μl ^15^N labeled (NH_4_)_2_SO_4_ (N mineralization and NH_4_^+^ uptake) or KNO_3_ (nitrification) to duplicates of 2 g of field-moist soil (0.125 mM, 10 at%, Sigma–Aldrich). Samples were incubated for 4 h and 24 h at 7 °C, extracted with 13 ml of 2 M KCl for 30 min, and filtered through ashfree filter paper (Whatman 40 ashfree cellulose filter). To stabilize the extracts, 20 μl 5 mM phenylmercuric acetate were added, and samples were frozen until further processing. For N mineralization and NH_4_^+^ uptake, NH_4_^+^ was diffused into acid traps and measured with an EA-IRMS system consisting of a CE Instrument EA 1110 elemental analyzer coupled to a Finnigan MAT DeltaPlus IRMS with a Finnigan MAT ConFlo II Interface. For nitrification, NH_4_^+^ was removed from the samples and NO_3_^−^ converted to NH_4_^+^ before diffusion into acid traps and EA-IRMS analysis (Mooshammer et al., 2012). Gross rates were based on differences in concentration and isotopic composition of NH_4_^+^, NO_3_^−^ or amino acids between two time points (e.g., 4 h and 24 h) and were calculated according to the equations described in Wanek et al. (2010).

As an indicator for microbial N limitation, we calculated the efficiency of microorganisms to use amino acid N for biomass growth (N use efficiency, NUE) by comparing gross rates of amino acid uptake and N mineralization:

NUE = (gross amino acid uptake − gross N mineralization)/gross amino acid uptake.

### 2.4 Phospholipid fatty acid (PLFA) analysis

For analysis of PLFAs, samples were stored frozen (Greenland heath tundra) or in RNAlater (both Siberian sites; Schnecker et al., 2012). Phospholipid fatty acids were extracted from 1 g of soil with chloroform/methanol/citric acid buffer and purified on silica columns (LC-Si SPE, Supelco) using chloroform, acetone and methanol (Frostegård et al., 1991; with the modifications described by Kaiser et al., 2010). After addition of methyl-nonadecanoate as internal standard, PLFAs were converted to fatty acid methyl esters (FAMEs) by alkaline methanolysis. Samples were analyzed on a Thermo Trace GC with FID detection: 1 μl per sample was injected in splitless mode (injector temperature 230 °C) and separated on a DB-23 column (Agilent; GC method:70 °C for 1.5 min, 30 °C min^−1^ to 150 °C, 150 °C for 1 min, 4 °C min^−1^ to 230 °C, 230 °C for 15 min) with 1.5 ml min^−1^ He as carrier. Individual FAMEs were identified using qualitative standard mixes (37 Comp. FAME Mix and Bacterial Acid Methyl Esters CP Mix, Supelco) and quantified by comparison with the internal standard. We used 18:1ω9, 18:2ω6,9 and 18:3ω3,6,9 fatty acids as biomarkers for fungi, i15:0, a15:0, i16:0, i17:0 and a17:0 for gram positive bacteria, cy17:0 (9/10), cy19:0 (9/10), 16:1ω5, 16:1ω7, 16:1ω9 and 18:1ω7 for gram negative bacteria, and 14:0, 15:0, 16:0, 17:0, 18:0, 20:0, i14:0, 16:1ω10, 16:1ω11, 17:1ω6 and 10Me16:0 as unspecific markers (Kaiser et al., 2010).

### 2.5 Statistics

To test for significant differences between sites and horizons, we applied two-way ANOVAs and Tukey HSD tests (after transformation, if necessary) or Kruskal–Wallis tests with unpaired Mann Whitney *U* tests as post-hoc tests if normal distribution and homoscedasticity could not be achieved. We additionally performed a Principal Component Analysis including gross N transformation rates (per g total N), pH values and the relative abundance of microbial groups. Samples with missing values were omitted from the Principal Component Analysis. Correlations were tested using Spearman's rank correlations. All statistics were performed in R 2.15 (R Development Core Team, 2012).

## 3 Results

Organic C, total N, and total P, as well as the C/N ratio, decreased significantly from organic to cryoturbated and mineral horizons (Tables 1 and 2). pH-Values were in the range of 4.3–5.5 for organic horizons and significantly higher in cryoturbated and mineral horizons (5.1–6.1 and 5.3–6.4, respectively). Microbial biomass (estimated as the total amount of PLFAs per g DW) decreased significantly from organic to cryoturbated and mineral horizons (Supplementary Fig. 1, Table 2), mainly due to differences in SOM content.

**Fig. 1:**
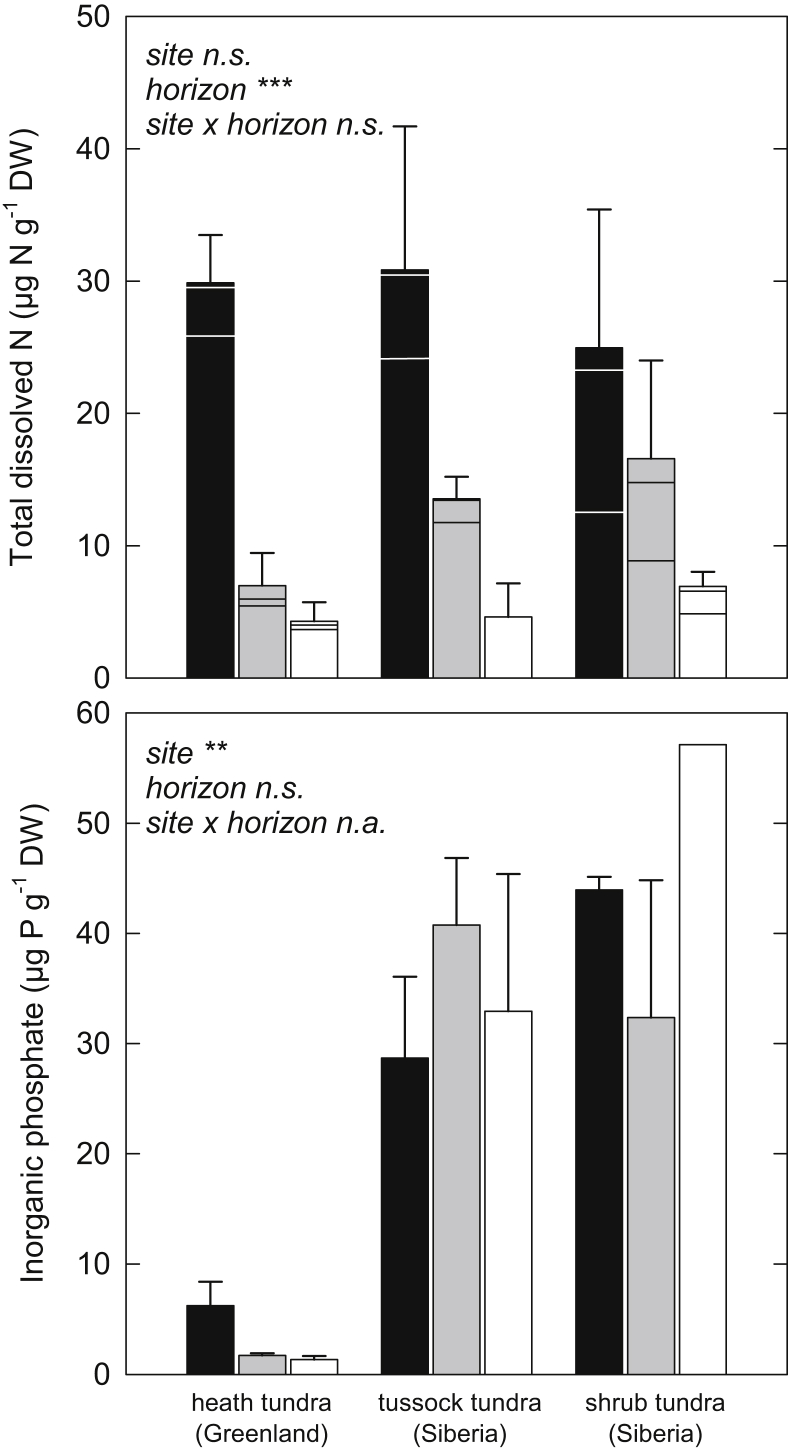
Upper panel: Total dissolved N in soil extracts of organic (black bars), cryoturbated (gray bars) and mineral (white bars) horizons of three tundra sites. Bars are separated into DON (lowest part), NH_4_^+^ (middle part) and NO_3_^−^ (upper part). Concentrations of NH_4_^+^ and DON could not be determined in the mineral horizons of the tussock tundra site. Lower Panel: Concentrations of inorganic phosphate in soil extracts of organic (black bars), cryoturbated (gray bars) and mineral (white bars) horizons of three tundra sites. All bars represent means ± standard error. Levels of significance: ***, *p* < 0.001; **, *p* < 0.01; *, *p* < 0.05; n.s., not significant; n.a., not analyzed (two-way ANOVA or Kruskal–Wallis test).

In all horizons, the pool of dissolved N was dominated by organic N (Fig. 1). The relative contribution of DON to the TDN pool was similar in all horizons at each site, although absolute concentrations were significantly lower in mineral than in organic horizons (Table 2). We did, however, observe significant variations in the composition of the TDN pool between sites. Dissolved organic N accounted for 85 ± 3% (mean ± standard error of all horizons) and 86 ± 5% of the TDN pool at the heath tundra and tussock tundra sites, but only for 58 ± 7% at the shrub tundra site (Fig. 1). Also concentrations of inorganic phosphate varied significantly between sites. Inorganic phosphate was lower by a factor of ten at the heath tundra site in Greenland than at both Siberian sites, but did not show significant differences between horizons (Fig. 1).

**Fig. 2:**
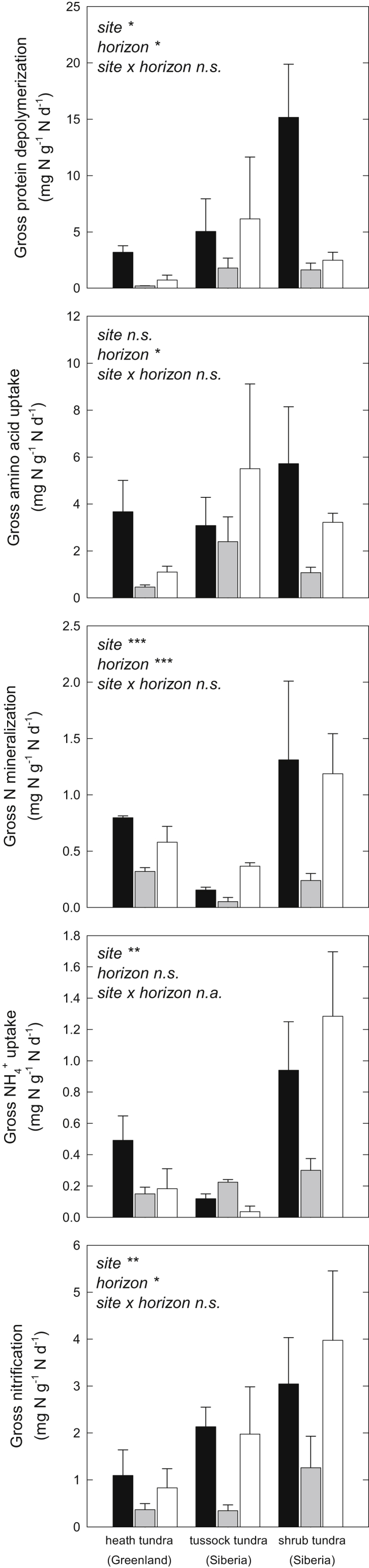
Gross rates of protein depolymerization, microbial amino acid uptake, N mineralization, microbial NH_4_^+^ uptake and nitrification (per g total N), of organic (black bars), cryoturbated (gray bars) and mineral (white bars) horizons of three tundra sites. Rates were measured using a set of ^15^N pool dilution approaches. Bars represent means ± standard error. Levels of significance: ***, *p* < 0.001; **, *p* < 0.01; *, *p* < 0.05; n.s., not significant; n.a., not analyzed (two-way ANOVA or Kruskal–Wallis test).

Gross rates of protein depolymerization, amino acid uptake, N mineralization, NH_4_^+^ uptake and nitrification, expressed per g DW, were generally highest in organic horizons, and decreased significantly from organic to cryoturbated and mineral horizons (Supplementary Fig. 2, Table 2). All N transformation rates were significantly and positively correlated with C and N content (Table 3). In order to assess horizon-specific differences in transformation rates that were not related to differences in SOM content, we calculated all gross N transformation rates per g total soil N. Cryoturbated horizons still exhibited significantly lower gross rates of protein depolymerization, amino acid uptake, N mineralization and nitrification per g total N than organic horizons, on average accounting for 16% (protein depolymerization), 32% (amino acid uptake), 27% (N mineralization) and 31% (nitrification) of the respective rates in organic horizons. In the case of N mineralization and nitrification, rates in cryoturbated horizons were even significantly lower than in mineral horizons (Fig. 2, Table 2).

In spite of the differences in absolute N transformation rates, NUE did not differ significantly between horizons (Table 2), and was not correlated with soil C/N ratio (*p* = 0.130, *R*^2^ = 0.10). We did, however, observe significant differences in NUE between sites. NUE was highest at the tussock tundra site in Siberia, where 90% of amino acid N taken up was incorporated into microbial biomass, and only 10% were mineralized to NH_4_^+^. NUE was lower at both other sites, with 66% incorporation at the shrub tundra site in Siberia, and 51% at the heath tundra site in Greenland (Fig. 4).

**Fig. 3:**
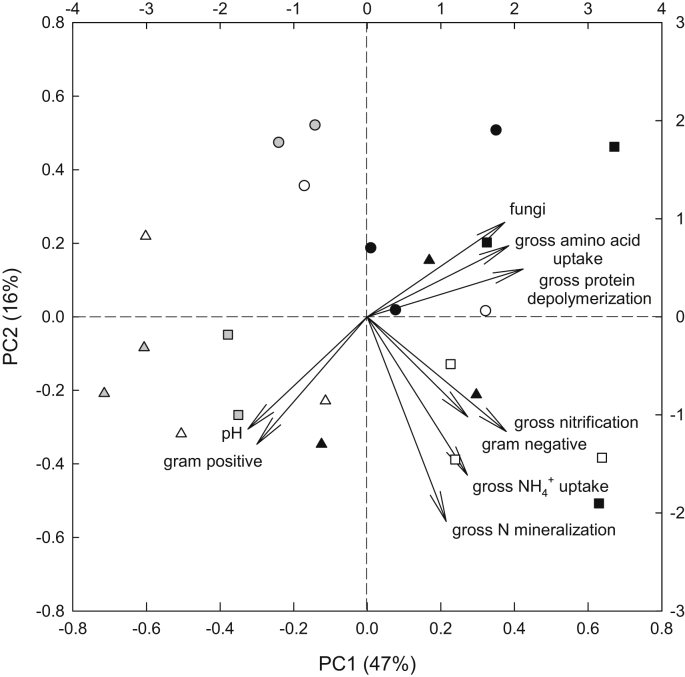
Ordination of organic (black), cryoturbated (gray) and mineral (white) horizons of heath tundra (Greenland; triangles), tussock tundra (Siberia; circles) and shrub tundra (Siberia; squares) using Principal Component Analysis. Data include gross rates of protein depolymerization, microbial amino acid uptake, N mineralization, microbial NH_4_^+^ uptake and nitrification (per g total N to correct for differences in SOM content between horizons), as well as pH values and the relative abundances of fungi, gram positive and gram negative bacteria (in % of total PLFAs).

To investigate possible relationships between N transformations and microbial community composition across horizons and sites, we performed a Principle Component Analysis, including gross N transformation rates (per g total N), the relative abundances of fungi, gram negative and gram positive bacteria (in % of total PLFAs), and pH values (Fig. 3). Principal Component 1 accounted for 47% of the variation in the data set, and was positively connected to all gross N transformation rates (factor loadings 0.42 for protein depolymerization, 0.38 for amino acid uptake, 0.21 for N mineralization, 0.27 for NH_4_^+^ uptake and 0.38 for nitrification), and thus represents an aggregated parameter of N transformation activity. Principal Component 1 separated all cryoturbated horizons and the mineral horizons of the Greenland heath tundra site from organic and the Siberian mineral horizons. These findings were supported by a two-way ANOVA that showed significantly lower values of Principal Component 1 for cryoturbated horizons compared to organic and mineral horizons, and for the Greenland heath tundra site compared to both Siberian sites (Table 2). Principal Component 2 (16% of variation), in contrast, was positively connected to gross rates of protein depolymerization and microbial uptake of amino acids (factor loadings 0.13 and 0.19), but negatively connected to gross rates of N mineralization, NH_4_^+^ uptake and nitrification (−0.55, −0.43 and −0.31), thus separating organic from inorganic N transformation processes. Principal Component 2 was also significantly correlated with NUE which reflects the allocation of amino acid N to mineralization (*p* = 0.004, *R*^2^ = 0.34).

The microbial groups of fungi, gram negative and gram positive bacteria contributed differently to Principal Components 1 and 2. Principal Component 1 was positively connected to the relative abundances of fungi and gram negative bacteria (factor loadings 0.37 and 0.27), and negatively to gram positive bacteria (−0.30). Principal Component 2 was positively connected to fungi (0.26) and negatively to both gram negative and gram positive bacteria (−0.27 and −0.35).

We further investigated possible relationships between individual N transformation processes and microbial groups using Spearman's rank correlations, and found that gross rates of protein depolymerization, amino acid uptake and nitrification (per g total N) were significantly correlated with the relative abundance of fungi, and that gross rates of amino acid uptake and nitrification were significantly correlated with gram negative bacteria (Table 3).

## 4 Discussion

High-latitude systems are usually characterized by low N availability that limits both plant and microbial growth (Hobbie et al., 2002; Sistla et al., 2012). Plants and microorganisms rapidly immobilize all reactive N forms small enough for uptake, in particular amino acids (Jones and Kielland, 2002; Näsholm et al., 2009) and oligo-peptides (Hill et al., 2011; Farrell et al., 2013), and incorporate the N into their biomass, with a minimum of microbial overflow mineralization to NH_4_^+^, and further transformation to NO_3_^−^. Although organic N forms represent a major source of N for both plants and microorganisms in the Arctic, we know little about the steps controlling their availability, i.e., protein depolymerization.

### 4.1 Nitrogen cycling in cryoturbated horizons

In the Arctic soils studied, gross rates of protein depolymerization, but also microbial amino acid uptake, N mineralization and nitrification were significantly lower in cryoturbated than in organic horizons, on average accounting for only 26% of the rates in organic horizons (Fig. 2). This corroborates our hypothesis that the whole sequence of N transformations, starting with the rate-limiting step of protein depolymerization, is decelerated in cryoturbated compared to organic horizons.

Protein depolymerization limits the amount of amino acids available for microbial uptake, and with it the potential for microbial growth and N mineralization. Severe N deficiency can even limit the production of extracellular enzymes that depolymerize complex organic compounds (Weintraub and Schimel, 2005a; Wallenstein et al., 2009; Sistla et al., 2012), including N-containing macromolecules such as proteins. While we did not directly estimate N availability in cryoturbated horizons, lower protein depolymerization rates compared to organic horizons point to a reduced N availability in cryoturbated horizons. We therefore suggest that the slow decomposition of cryoturbated SOM (Kaiser et al., 2007; Xu et al., 2009; Hugelius et al., 2010) might be connected to N limitation of enzyme production. In this case, an increase in protein depolymerization with climate change (Weedon et al., 2011; Brzostek et al., 2012) could facilitate the decomposition of cryoturbated SOM, and lead to higher CO_2_ emissions from Arctic soils.

### 4.2 Microbial communities and nitrogen transformations

The differences in N cycling across soil horizons and sites were likely caused, at least in part, by differences in composition and N demand of the microbial communities. The rate-limiting step in N cycling, protein depolymerization, requires specific enzymes, i.e., proteases, that are produced by a range of bacteria and fungi. We here found that gross protein depolymerization rates were significantly correlated with the relative abundance of fungi (Table 3). Fungi are able to produce a wide range of extracellular enzymes (Baldrian et al., 2011; Schneider et al., 2012) and are involved in the degradation of many complex organic molecules including cellulose and lignin (de Boer et al., 2005; Strickland and Rousk, 2010). Protein breakdown in particular has been assigned to ecto- and ericoid mycorrhizal fungi in high latitude systems (Read and Perez-Moreno, 2003). A low abundance of fungi in cryoturbated horizons might thus not only contribute to the low protein depolymerization rates, but also generally to the retarded decomposition of cryoturbated SOM.

Protein depolymerization was closely correlated with microbial amino acid uptake (Table 3). We found a similar close correlation for N mineralization and NH_4_^+^ uptake. This suggests that the microbial uptake of amino acids and NH_4_^+^ was limited by the respective production rates (protein depolymerization and N mineralization), indicating a high demand of the microbial biomass for N. A tight coupling of production and consumption rates of both amino acids and NH_4_^+^ has already been demonstrated for decomposing beech leaf litter (Mooshammer et al., 2012).

While N mineralization obviously limited the amount of NH_4_^+^ available for microbial uptake, N mineralization itself was not correlated with any upstream process such as protein depolymerization or amino acid uptake, or with any microbial group (Table 3). Nitrogen mineralization is the microbial de-amination of organic N and excretion of NH_4_^+^. The potential for N mineralization thus depends on the uptake of organic N by microorganisms. Microorganisms, however, can directly control the amount of N mineralized, and will only mineralize an excess of N that is not needed for growth or other cellular processes (Schimel and Bennett, 2004). Actual N mineralization rates therefore reflect both N availability and N demand for growth by the microbial community.

Nitrification, in contrast, was significantly correlated with the relative abundances of gram negative bacteria and fungi (Table 3). Nitrification requires a specific set of enzymes that oxidize ammonium over nitrite to nitrate, and is restricted to specific microbial groups. Autotrophic nitrification was found in few groups of archaea and gram negative bacteria (Hayatsu et al., 2008; Schleper, 2010; Alves et al., 2013). The abundance of gram negative bacteria might therefore influence nitrification rates, as already demonstrated for savanna and forest soils (Balser and Firestone, 2005). Heterotrophic nitrification, in contrast, is more widespread among microorganisms, but was mainly connected to fungi, particularly in acidic soils (Hayatsu et al., 2008).

### 4.3 Nitrogen use efficiency of the microbial community

**Fig. 4:**
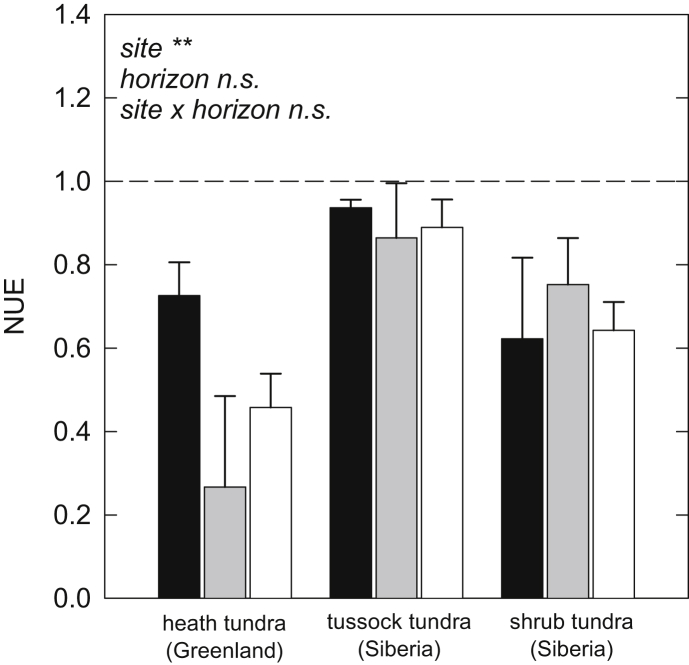
Nitrogen use efficiency (NUE) of organic (black bars), cryoturbated (gray bars) and mineral (white bars) horizons of three tundra sites. NUE was calculated as the proportion of amino acid N taken up by microorganisms that was not mineralized to NH_4_^+^. Bars represent means ± standard error. Levels of significance: ***, *p* < 0.001; **, *p* < 0.01; *, *p* < 0.05; n.s., not significant (two-way ANOVA).

In addition to low protein depolymerization rates in cryoturbated horizons, the observed reduction in N mineralization may also have been caused by a higher allocation of the available N to growth, and thus by a higher NUE. Our results demonstrate, however, that microbial NUE did not differ significantly between horizons (Fig. 4), indicating that the microbial communities in all horizons, including the cryoturbated ones, had a similar demand for N. Furthermore, NUE was not correlated with soil C/N ratios, suggesting that the decrease in C/N ratio from organic to mineral horizons (Table 1) was likely offset by changes in C and N availability with soil depth. Montané et al. (2007) showed for a mountain grassland soil, that while persistence of soil C was constant across the soil profile, persistence of soil N increased with soil depth, probably due to differences in chemical composition or in binding to soil minerals (for a recent review on SOM persistence see Schmidt et al., 2011).

NUE was generally rather high (as expected for N-limited systems), but differed significantly between sites (Fig. 4). NUE was significantly higher and gross N mineralization significantly lower at the tussock tundra site in Siberia than at the heath tundra site in Greenland, indicating that the microbial community at the tussock tundra site needed a higher proportion of the available N for growth. Size and composition of the TDN pool, as well as gross protein depolymerization and amino acid uptake rates were similar at both sites (Table 2), so it can be assumed that N availability for microorganisms was similar – pointing to the fact that N was not the main limiting element at the Greenland heath tundra site.

Since concentrations of inorganic phosphate were significantly lower at the Greenland heath tundra than at both Siberian sites (Fig. 1), we suggest that microbial growth at the Greenland heath tundra site could have been rather limited in P. Microorganisms have to maintain a rather constrained C:N:P ratio and have only limited capacities to store excess C, N or P in the biomass. Therefore, P limitation should lead to increased N mineralization as an overflow mechanism (Tezuka, 1990; Sterner and Elser, 2002). Although P limitation is usually considered characteristic for old, highly weathered soils, for example in the tropics (Cleveland et al., 2002), there is increasing evidence that P is an important co-limiting and in some cases even the main limiting element in Arctic soils (Shaver and Chapin, 1995; Shaver et al., 1998; Giesler et al., 2002; Hartley et al., 2010; Giesler et al., 2012). In the case of the heath tundra site, we found that although concentrations of inorganic phosphate were significantly lower than at the Siberian sites, total P content was similar, indicating that differences in the sorption of P to mineral soil particles may have been responsible for the low P availability at this site.

### 4.4 Conclusions

We found significant differences in N transformation rates and microbial N use efficiency between sites and horizons, demonstrating that the respective microbial communities differed in nutrient limitation. Across all sites, N cycling was slower in cryoturbated compared to organic horizons, starting with protein depolymerization, which is rate-limiting in N cycling. Our results thus indicate that microbial communities have a lower capacity to break down proteins in cryoturbated compared to organic horizons, likely due to differences in community composition (e.g., a lower abundance of fungi). Overall, our study suggests that burial of organic material by cryoturbation into the subsoil leads to changes in soil N transformations, which in turn may contribute to the observed retarded decomposition of cryoturbated SOM by altering N availability for microbial decomposers.

## Figures and Tables

**Table 1:** C, N and P content, C/N and N/P ratios (mass ratios), and pH values of organic, cryoturbated (cryot.) and mineral horizons of heath tundra (Greenland), tussock tundra (Siberia) and shrub tundra (Siberia). Values represent means (±standard error).

Site	Horizon	C (%)	N (%)	P (%)	C/N	N/P	pH
Heath	Organic	15.76 (0.86)	0.93 (0.06)	0.102 (0.006)	17.14 (1.95)	9.18 (0.39)	5.29 (0.10)
	Cryot.	9.35 (0.95)	0.65 (0.00)	0.112 (0.007)	14.46 (1.46)	5.82 (0.37)	6.02 (0.02)
	Mineral	3.94 (1.01)	0.29 (0.08)	0.074 (0.025)	13.81 (0.61)	4.86 (1.57)	6.16 (0.11)
Tussock	Organic	22.15 (1.57)	1.00 (0.05)	0.155 (0.019)	22.38 (2.18)	6.68 (1.09)	5.13 (0.05)
	Cryot.	15.30 (4.84)	0.81 (0.18)	0.112 (0.011)	18.23 (1.76)	7.36 (1.81)	5.57 (0.28)
	Mineral	2.08 (0.40)	0.14 (0.02)	0.046 (0.039)	14.33 (0.40)	9.40 (7.42)	5.50 (0.02)
Shrub	Organic	26.63 (5.98)	0.96 (0.18)	0.115 (0.005)	27.46 (1.61)	8.36 (1.57)	4.64 (0.23)
	Cryot.	9.69 (2.57)	0.64 (0.20)	0.121 (0.084)	15.46 (0.88)	8.01 (3.90)	5.80 (0.29)
	Mineral	2.50 (0.85)	0.16 (0.03)	0.061 (0.022)	14.66 (2.96)	4.55 (2.66)	5.54 (0.19)

**Table 2:** Significance of differences between sites (heath tundra, Greenland; tussock tundra, Siberia; shrub tundra, Siberia) or between horizons (organic; cryoturbated; mineral), derived from two-way ANOVA with Tukey HSD test, or Kruskal–Wallis tests with Mann–Whitney *U* tests as post-hoc. Different letters indicate *p* < 0.05, with “a” denoting the highest values.

Parameter	Unit	Between sites				Between horizons			
		Sign.	Heath	Tussock	Shrub	Sign.	Organic	Cryot.	Mineral
C	%	n.s.				***	a	b	c
N	%	n.s.				***	a	a	b
P	%	n.s.				*	a	ab	b
C/N	g C g^−1^ N	n.s.				***	a	b	b
N/P	g N g^−1^ P	n.s.				n.s.			
pH		**	a	b	b	***	b	a	a
Total PLFAs	μmol g^−1^ DW	n.s.				***	a	b	c
TDN	μg N g^−1^ DW	n.s.				***	a	b	b
DON	μg N g^−1^ DW	n.s.				**	a	b	b
NH_4_^+^	μg N g^−1^ DW	*	b	ab	a	*	a	ab	b
NO_3_^-^	μg N g^−1^ DW	n.s.				n.s.			
DON	% of TDN	**	a	a	b	n.s.			
Inorganic phosphate	μg P g^−1^ DW	**	b	a	a	n.s.			
Gross protein depolymerization	μg N g^−1^ DW d^−1^	n.s.				***	a	b	b
Gross amino acid uptake	μg N g^−1^ DW d^−1^	n.s.				***	a	b	b
Gross N mineralization	μg N g^−1^ DW d^−1^	***	a	b	a	***	a	b	b
Gross NH_4_^+^ uptake	μg N g^−1^ DW d^−1^	*	ab	b	a	***	a	a	b
Gross nitrification	μg N g^−1^ DW d^−1^	n.s.				**	a	b	b
Gross protein depolymerization	mg N g^−1^ N d^−1^	*	b	ab	a	*	a	b	ab
Gross amino acid uptake	mg N g^−1^ N d^−1^	n.s.				*	a	b	ab
Gross N mineralization	mg N g^-1^ N d^−1^	***	a	b	a	***	a	b	a
Gross NH_4_^+^ uptake	mg N g^−1^ N d^−1^	**	b	b	a	n.s.			
Gross nitrification	mg N g^−1^ N d^−1^	**	b	ab	a	*	a	b	a
NUE		**	b	a	b	n.s.			
Principal Component 1		***	b	a	a	***	a	b	a
Principal Component 2		*	b	a	b	n.s.			

Levels of significance: ***, *p* < 0.001; **, *p* < 0.01; *, *p* < 0.05; n.s., not significant.

**Table 3:** Correlation analysis of soil C and N content (in % of DW), microbial abundances (in % of total PLFAs) and gross rates of protein depolymerization (Protein depol.), microbial amino acid uptake (AA uptake), N mineralization, microbial NH_4_^+^ uptake and nitrification, measured with a set of ^15^N pool dilution approaches. For correlation with soil C and N content, gross N transformation rates were expressed in μg N g^−1^ DW d^−1^. For correlation with microbial groups, and with each other, gross N transformation rates were corrected for the differences in SOM content between soil horizons and expressed in mg N g^−1^ N d^−1^. Significance of correlations and correlation coefficients were determined using Spearman's rank correlations.

	Protein depol.	AA uptake	N mineralization	NH_4_^+^ uptake	Nitrification
C	+0.577**	+0.621**	+0.445*	+0.565**	+0.639**
N	+0.519*	+0.541**	+0.418*	+0.539**	+0.650**
Fungi	+0.499*	+0.540**	+0.367	+0.176	+0.428*
Gram negative	+0.368	+0.483*	+0.304	+0.126	+0.528*
Gram positive	−0.305	−0.396	−0.166	−0.092	−0.276
AA uptake	+0.792***				
N mineralization	+0.237	+0.264			
NH_4_^+^ uptake	+0.356	+0.244	+0.532**		
Nitrification	+0.597**	+0.399	+0.424*	+0.394	

Levels of significance: ***, *p* < 0.001; **, *p* < 0.01; *, *p* < 0.05.
