# Supplementary material for: Nitrogen dynamics in Turbic Cryosols from Siberia and Greenland
Source: Soil Biol Biochem. 2013 Dec;67:85–93. doi: 10.1016/j.soilbio.2013.08.004 (PMC3819518; doi:10.1016/j.soilbio.2013.08.004)
Supplement: Supplementary file 1 [file mmc1.docx]

Supplementary Fig. 1. Concentrations of phospholipid fatty acids (PLFAs) in organic (black bars), cryoturbated (grey bars) and mineral (white bars) horizons of three tundra sites. Bars represent means ± standard error. Levels of significance: ***, p < 0.001; **, p < 0.01; *, p < 0.05; n.s., not significant (two-way ANOVA).

Supplementary Fig. 2. Gross rates of protein depolymerization, microbial amino acid uptake, N mineralization, microbial NH_4_^+^ uptake and nitrification (per g dry weight), of organic (black bars), cryoturbated (grey bars) and mineral (white bars) horizons of three tundra sites. Rates were measured using a set of ^15^N pool dilution approaches. Bars represent means ± standard error. Levels of significance: ***, p < 0.001; **, p < 0.01; *, p < 0.05; n.s., not significant (two-way ANOVA).
